# Retraction Note: Treatment for lumbar spinal stenosis secondary to ligamentum flavum hypertrophy using percutaneous endoscopy through interlaminar approach: a retrospective study

**DOI:** 10.1186/s13018-021-02593-1

**Published:** 2021-07-08

**Authors:** Yi Liu, Yingjie Qi, Diarra Mohamed Diaty, Guanglei Zheng, Xiaoqiang Shen, Shangben Lin, Jiaqi Chen, Yongwei Song, Xiaomin Gu

**Affiliations:** Department of Orthopaedics, The Affiliated Hospital of Hangzhou Normal University, 126 Wenzhou Road, Hangzhou, 310000 China

**Retraction Note: J Orthop Surg Res 15, 337 (2020)**

**https://doi.org/10.1186/s13018-020-01874-5**

The Editor-in-Chief has retracted this article because after publication the authors informed the Journal that, contrary to the information in the article, all patients in the control group had received lumbar spine fusion surgery. The Editor-in-Chief therefore considers that comparison between the control group and the endoscopy group is not valid. Xiaomin Gu agrees with this retraction. Yi Liu, Yingjie Qi, Diarra Mohamed Diaty, Guanglei Zheng, Xiaoqiang Shen, Shangben Lin, Jiaqi Chen and Yongwei Song have not responded to any correspondence from the Editor-in-Chief or the publisher about this retraction.

